# Uncovering a Novel Functional Interaction Between Adult Hepatic Progenitor Cells, Inflammation and EGFR Signaling During Bile Acids-Induced Injury

**DOI:** 10.7150/ijbs.90645

**Published:** 2024-04-08

**Authors:** Juan García-Sáez, María Figueroa-Fuentes, Carlos González-Corralejo, Cesáreo Roncero, Nerea Lazcanoiturburu, Álvaro Gutiérrez-Uzquiza, Javier Vaquero, Ester González-Sánchez, Kunzangla Bhutia, Silvia Calero-Pérez, Flavio Maina, Javier Traba, Ángela M. Valverde, Isabel Fabregat, Blanca Herrera, Aránzazu Sánchez

**Affiliations:** 1 Dept. of Biochemistry and Molecular Biology, Faculty of Pharmacy, Complutense University of Madrid (UCM), Health Research Institute of the “Hospital Clínico San Carlos” (IdISSC), Madrid, Spain.; 2 TGF-β and Cancer Group, Oncobell Program, Bellvitge Biomedical Research Institute (IDIBELL), Barcelona, Spain.; 3 Biomedical Research Networking Center in Hepatic and Digestive Diseases (CIBEREHD-ISCIII), Madrid, Spain.; 4 Dept. Biochemistry and Molecular Biology, Faculty of Biology, Complutense University of Madrid (UCM), Madrid, Spain.; 5 Biomedical Research Institute Sols-Morreale, Spanish National Research Council and Autonomous University of Madrid (IIBM, CSIC-UAM); Biomedical Research Networking Center in Diabetes and Associated Metabolic Disorders of the Carlos III Health Institute (CIBERdem-ISCIII), Madrid, Spain.; 6 Aix Marseille Univ, Inserm, CNRS, Centre de Recherche en Cancérologie de Marseille (CRCM), Institut Paoli-Calmettes, Turing Center for Living Systems, Marseille, France.; 7 Dept. for Molecular Biology, Center for Molecular Biology Severo Ochoa, Spanish National Research Council-Autonomous University of Madrid (CSIC-UAM), Madrid, Spain.

**Keywords:** EGFR, Bile acids, Hepatic Progenitor Cell, Inflammation, Secretome, Liver Disease

## Abstract

Chronic cholestatic damage is associated to both accumulation of cytotoxic levels of bile acids and expansion of adult hepatic progenitor cells (HPC) as part of the ductular reaction contributing to the regenerative response. Here, we report a bile acid-specific cytotoxic response in mouse HPC, which is partially impaired by EGF signaling. Additionally, we show that EGF synergizes with bile acids to trigger inflammatory signaling and NLRP3 inflammasome activation in HPC. Aiming at understanding the impact of this HPC specific response on the liver microenvironment we run a proteomic analysis of HPC secretome. Data show an enrichment in immune and TGF-β regulators, ECM components and remodeling proteins in HPC secretome. Consistently, HPC-derived conditioned medium promotes hepatic stellate cell (HSC) activation and macrophage M1-like polarization. Strikingly, EGF and bile acids co-treatment leads to profound changes in the secretome composition, illustrated by an abolishment of HSC activating effect and by promoting macrophage M2-like polarization. Collectively, we provide new specific mechanisms behind HPC regulatory action during cholestatic liver injury, with an active role in cellular interactome and inflammatory response regulation. Moreover, findings prove a key contribution for EGFR signaling jointly with bile acids in HPC-mediated actions.

## Introduction

Chronic liver diseases (CLDs) represent a major global public health problem, with a mortality rate of approximately 2 million deaths per year worldwide 1. Among CLD, the cholestatic liver disorders are characterized by a blockage or marked reduction in bile flow primarily caused by either a functional impairment of hepatocytes or cholangiocytes for bile secretion or by obstruction of bile flow through bile ducts 2, 3. Pathological mechanisms behind the cholestatic liver injury are not well understood. Nevertheless, the intrahepatic accumulation of bile acids at supraphysiological levels constitutes a major cause of toxic injury in liver parenchyma 3-5, which may progress to biliary fibrosis associated or not with cirrhosis, and eventually, to end-stage liver disease. Similarly to other CLDs, an inflammatory response plays an important role in cholestatic disorders 6-8. In this sense, regardless of their intrinsic toxicity, bile acids have emerged as signaling molecules participating in this inflammatory response, been able to stimulate secretion of cytokines and chemokines in hepatocytes and cholangiocytes, possibly contributing to the inflammatory misregulation and disease progression 9-11.

Cholestatic injury is also characterized by a ductular reaction, a term referring to the appearance of proliferating intrahepatic bile ductular structures sprouting into periportal and parenchymal regions 12. The origin of these structures is not fully clear and vary depending on the etiology of injury 13. Nevertheless, proliferation of bipotential hepatic progenitor cells (HPC), together with other hepatic cells, particularly inflammatory and stellate cells, create a cellular network impacting the pathophysiology of cholestatic liver diseases. HPC constitute an alternative source for generating hepatocytes and cholangiocytes when mature cells lose their replicative and functional capacity 14-16. Still, they play a key role in CLD development and evolution, having both a pro-regenerative and a pro-fibrogenic potential 17, 18 with a delicate context-dependent balance. Remarkably, despite their concurrency in the cholestatic liver, little is known about HPC response to bile acids.

In the present study, we characterize the mouse HPC response to cholestatic injury and clarify whether this cell population has an active role in the inflammatory response induced during cholestatic injury, with a special focus in understanding cell communication between HPC and other hepatic cell populations relevant in the cholestatic liver. Additionally, we evaluate the specific contribution of EGFR signaling in this scenario. The EGFR signaling pathway is a central pathway in liver regeneration 19, and an important regulator of HPC biology 20, 21, but its specific actions in cholestatic liver diseases remain ambiguous. Indeed, a protective role for EGFR activity was proposed during cholestatic injury developed in *Mdr2*^-/-^ mice 22. However, we and others have shown that a diminished EGFR signaling in hepatocytes improves the regenerative response in the 3,5-diethoxycarbonyl-1,4-dihydrocollidine (DDC)-supplemented diet model of cholestatic liver injury 22, 23. This agrees with data evidencing an attenuation of biliary fibrosis upon EGFR inhibition in the bile duct ligation (BDL) model 24. Furthermore, we have demonstrated that EGFR activity is critical in the crosstalk between parenchymal and non-parenchymal hepatic cells, promoting the pro-inflammatory response activated during cholestatic injury 23. Whether or not the HPC population plays a part in such regulatory crosstalk was not determined. All this prompted us to try to elucidate a potential functional interaction axis between HPC, inflammatory response and EGFR signaling in a context of cholestatic injury.

## Material and Methods

### Cell Lines and Culture Conditions

HPC lines were generated as described 25 and maintained in Dulbecco's modified Eagle's (DMEM) medium supplemented with 10% fetal bovine serum (FBS). Immortalized mouse hepatocytes were generated as described 26, 27 and cultured in Williams´ medium E supplemented with 10% FBS, 2mM glutamine, 0.75mM sodium pyruvate and 0.4 µg/mL dexamethasone. Primary mouse hepatocytes were isolated following the two-step collagenase perfusion technique followed by isodensity purification in a percoll gradient 28. Briefly, livers from two-three-month-old male mice were perfused with Hank's balanced salt solution supplemented with 10mM Hepes and 0.2mM EGTA for 5 min, followed by 15 min perfusion with Williams´ medium E containing 10mM Hepes and 0.03% collagenase type I (125 U/mg; Worthington). Livers were further minced, cell suspension was filtered through a 70μm cell strainer (BD) and viable hepatocytes were selected by centrifugation in Percoll, and seeded in collagen I-coated plates at a density of 25,000 cells/cm^2^ in DMEM:F12 (1:1) supplemented with 10% FBS. Cells were kept overnight at 37ºC in 5% CO_2_ and used 12-16 h later. Immortalized mouse HSC line GRX 29 was maintained in 10% FBS DMEM.

For all cell lines (HPC, immortalized hepatocytes and HSC) medium was replaced every three days, and cells were subcultured at 80%-90% confluence.

Mouse macrophages were isolated from the peritoneal cavity. Three months-old male mice were injected intraperitoneally with 2 mL 3% thioglycolate (BD GibcoTM). Four-five days later mice were euthanized and 10 mL of cold PBS were injected in the peritoneum, abdomen was gently massaged and then the peritoneal exudate fluid containing cells was aspirated slowly and centrifuged for 5 min at 1500 g and 4ºC. Cell pellet was resuspended in a 15.5mM NH_4_Cl, 12mM NaHCO_3_, 0.1mM EDTA lysis buffer to remove blood cells. Incubation was stopped after 90 seconds by adding 10% FBS DMEM and cells were centrifuged at 1500 g, 5 min at 4ºC and then plated in 10% FBS DMEM. Twenty-four hours later medium was replaced by DMEM with 2% FBS, and 24 h later, cells were serum starved and treated.

All cells were maintained in a humidified incubator at 37ºC and a 5% CO_2_ atmosphere.

The bile salts sodium taurochenodeoxycholate (TCDC), sodium glycochenodeoxycholate (GCDC), sodium taurocholate (TCA) and sodium chenodeoxycholate (CDC) and cholestatic agent 1-naphyhyl isothiocyanate (ANIT), were all purchased in Sigma Aldrich (Massachusetts, USA). Mouse bile was extracted from mice submitted to bile duct ligation for three weeks 30. EGF was from Peprotech (New Jersey, USA) and HB-EGF from R&D Systems (Minnesota, USA). Gefitinib was from Merck (Darmstadt, Germany) and SB431542 from Sigma Aldrich.

All animal procedures were done conformed to European Union Directive 86/609/EEC and Recommendation 2007/526/ EC, enforced in Spanish law under RD 1201/2005. Animal protocols were approved by the Animal Experimentation Ethics Committee of the UCM and the Animal Welfare Division of the Environmental Affairs Council of the Government of Madrid (Proex 326/15; 262.6/21 and 022.4/2023).

### Preparation of HPC conditioned medium

HPC were seeded at high density (34,000 cells/cm^2^). HPC were serum starved and medium was collected after 24 h. When HPC were treated, 2-3 h after serum starvation, EGF or bile acids were added for 2 h and then medium was replaced with serum-free and growth factor/bile acid-free fresh medium. For combined treatment, after 2 h of EGF stimulation, bile acids were added for 2 h and then medium was replaced with serum-free and growth factor/bile acid-free fresh medium. In all cases, after 24 h culture medium was collected, filtered (0.2μm pore size), centrifuged (800 g, 5 min, 4ºC), and stored at -20 ºC**.**

### RNA isolation, Quantitative and Reverse Transcriptase-Polymerase Chain Reaction (RT-qPCR)

Total cellular RNA was isolated using the NucleoSpin RNA kit (Macherey-Nagel). RNA yield and purity were analysed using a Nanodrop (NanoDrop 2000, Thermo Fisher Scientific, Waltham, MA, USA). RT-qPCR was performed as described before 21. Primers used in the study are listed in Supplementary Table S1.

### Cell viability assays

Quantification of cell number was performed as described 31. Cells were serum starved for 2-4 h prior to treatment with different factors. At different time points, cells were harvested by trypsinization and viable cells were counted using trypan blue staining and a Neubauer chamber. As additional approach, viable adherent cells were stained, as described 32, with crystal violet (Sigma-Aldrich, Sant Louis, Missouri). The absorbance of each plate was read photometrically at 590 nm using a plate reader (Powerwave XS, Biotek). Percentage of remaining viable cells was calculated with respect to control (untreated) cells.

MTT assay was performed according to manufacturer´s recommendations. Briefly, 20 µl of 3-(4,5-dimethylthiazol-2-yl)-2,5-diphenyltetrazole bromide (MTT) reagent (Promega) was added to a volume of 180 µl of culture medium, and the plate was incubated for 2 h at 37°C. MTT reduction for formazan was measured at 620 and 492 nm (wavelengths corresponding to reagent and product of the reduction reaction) in a plate reader (Powerwave XS, Biotek). Difference between the absorbance of the product and reagent for each well was calculated.

### Lactate dehydrogenase (LDH) assay

To measure the enzymatic activity of lactate dehydrogenase (LDH) in culture conditions, the medium was collected, and cells were lysed in a buffer containing 0.1M K_2_HPO_4_ 0.5% Triton X-100 pH 7.4. Reaction mixture contained culture medium (50 μL) or cell lysate (25 µL), 0.1M K_2_HPO_4_ buffer pH 7.4 (50 μL or 75 µL in the case of cell lysate), 80mM pyruvate and 2 mg/mL NADH. Finally, the absorbance at 340 nm was read for 20 min in a plate reader (Powerwave XS, Biotek). The enzyme activity in each fraction (culture medium and cell lysate) was expressed in U/μg. Percentage of LDH released corresponds to the enzyme activity in the medium relative to total activity of each sample.

### Protein isolation and western blot analysis

Total protein extracts from cultured cells were prepared in RIPA buffer (10mM Tris pH 7.5; 150mM NaCl; 1% NP40; 2mM EDTA; 0,1% SDS; 1% sodium deoxycholate) supplemented with 1mM phenylmethylsulfonyl fluoride, 10 µg/mL aprotinin and leupeptin, 1mM sodium orthovanadate and 20mM sodium fluoride; or Laemmli buffer (125mM Tris, 4% SDS, 20% glycerol, 5% β-mercaptoethanol). Western blotting procedures were carried out as previously described 25, 33. 30 to 100 µg of protein were separated in 8-12% acrylamide SDS-polyacrylamide electrophoresis gels and blotted to PVDF membranes (Amersham Biosciences, Amersham, UK). Membranes were probed with the primary antibodies diluted as indicated in Supplementary Table S2 in Tris-buffered saline containing 0.1% Tween 20 and 0.5% non-fat dried milk or 0.5% bovine serum albumin according to manufacturer's instructions. Detection was performed using an enhanced chemiluminescence method in a Gel documentation system Imager CHEMI Premium (VWR International Eurolab SL, Linars del Vallès, Barcelona, Spain).

### Caspase-3-Like Enzymatic Activity

A fluorometric assay in the presence of Ac-DEVD-AMC as fluorogenic caspase-3 substrate was used following a previously described protocol 34. Reaction mixture contained 12.5 μL cell lysate, 162.5 μL assay buffer (20mM HEPES pH 7.5, 10% glycerol, 2mM DTT) and 20μM caspase-3 substrate (Ac-DEVD-AMC, from BD Biosciences). After 1 h incubation in the dark, fluorescence intensity was measured in a Microplate Fluorescence Reader (Tecan infinity 200®, 380/440 nm). We define a unit of caspase-3 activity as the amount of active enzyme necessary to produce an increase in 1 arbitrary unit in the fluorimeter after 1 h incubation reaction. Protein concentration of cell lysates was determined by using the BCA protein assay kit and results are expressed as units of caspase-3 activity per μg of protein.

### Caspase-1-Like Enzymatic Activity

A fluorometric assay in the presence of Z-YVAD-AFC as fluorogenic caspase-1 substrate was performed. Cells were lysed in 25mM Hepes, 5mM EGTA, 5mM DTT, pH 7.5, sonicated 30 sec and then centrifuged at 13,000 g for 5 min. Reaction mixture contained 50 μL cell lysate, 50 μL assay buffer (200mM HEPES, 0.2% CHAPS, 20% Sacarose, 29mM DTT pH 7.5) and 5μM caspase-1 substrate (Z-YVAD-AFC, from Enzo Life Sciences). After 30 min incubation in the dark, fluorescence intensity was measured in a Microplate Fluorescence Reader (Tecan infinity 200®, 400/505 nm). We define a unit of caspase-1 activity as the amount of active enzyme necessary to produce an increase in 1 arbitrary unit in the fluorimeter after 30 min incubation reaction. Then, protein concentration of cell lysates was determined by using the BCA protein assay kit and results are expressed as units of caspase-1 activity per μg of protein.

### Confocal microscopy analysis

For protein analysis by confocal fluorescence microscopy, we followed protocols previously described 35. Cells were seeded on 2% gelatin-coated glass coverslips in 10% FBS DMEM, serum staved and treated. Then, cells were fixed with 4% paraformaldehyde in PBS for 20 min at 4ºC, and permeabilized with 0.1% SDS 0.5% triton X-100. Blocking was performed in 3% BSA 1.5% Normal Goat Serum (NGS) in PBS for 1 h at RT. P65 monoclonal antibody (sc-8008 Santa Cruz Biotechnology) was diluted 1:100 in 3% BSA and 1.5% NGS in PBS and applied for 15h at 4ºC. Anti-mouse Alexa 488-conjugated secondary antibody (A11012, Invitrogen) was diluted 1:200 in PBS 3% BSA and 1.5% NGS and applied for 1h at RT together with DAPI (1:1000, from Sigma Merck). Antibody incubations were done in a humidity chamber to avoid evaporation. For visualization cells were embedded in ProLong™ Gold (Thermo Fisher) mounting medium and visualized in an Olympus FV1200 confocal microscopy.

### Proteomic analysis

Proteomic analysis was performed in the Proteomics Unit of the Complutense University of Madrid. A label-free experiment was conducted. Briefly, HPC conditioned medium samples were concentrated with speed vac and resuspended in 8M urea. Proteins were digested using an iST kit (Preomics, Planegg, Germany). The resulting peptides were analysed using liquid nano-chromatography (Vanquish Neo, Thermo Scientific), coupled to high-resolution mass spectrometer Q-Exactive HF (Thermo Scientific, Bremen, Germany). Proteins were identified using Proteome Discover 3.0 software (Thermo Scientific) and the search engine Mascot 2.6 (matrixscience.com). The database used was Uniprot (UP-000000589). For quantitative proteomics, the chromatograms and retention times of all samples were aligned. Afterwards, total protein abundance between different samples was normalized. Statistically, Student t-test was applied to assess proteins differential abundance between samples. A p-value < 0.05 was considered statistically significant. Gene ontology (GO) overrepresentation analysis of biological process terms was performed using clusterProfiler v4.4.2 36 and org.Hs.eg.db v3.14.0 (DOI: 10.18129/B9.bioc.org.Hs.eg.db). Redundant GO terms were excluded for graphical representation using the rrvgo v1.6.0 37. GO functional enrichment analysis of the secretory proteins was conducted on the website (http://geneontology.org/) using PANTHER Overrepresentation Test and GeneOntology database (DOI: 10.5281/zenodo.6799722) 38. The comparative proteomics data was used to perform gene set enrichment analysis (GSEA), using Molecular Signature Database gene sets (MSigDB v2023.1.Mm).

### Statistical analysis

Statistical analysis was performed by paired Student's t-test analysis or one-way ANOVA to calculate p-values once normal distribution of data was verified using Shapiro-Wilk test. For data with non-normal distribution, Kruskal Wallis test was used. A p-value < 0.05 was considered statistically significant.

## Results

### Bile acids and cholestatic agents induce cytotoxicity in hepatic progenitor cells, which is partially counteracted by EGFR signaling

**Figure 1:**
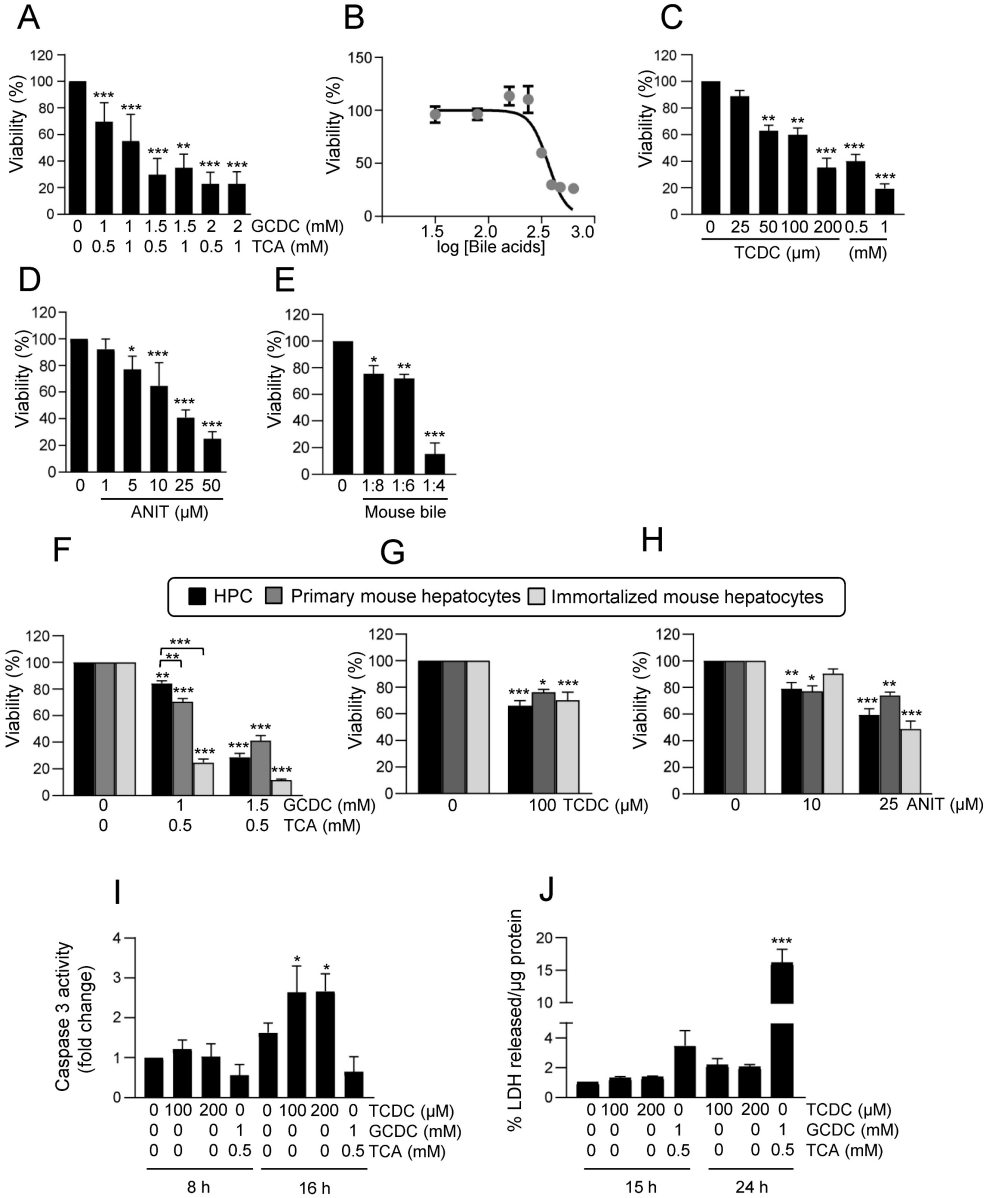
** Cytotoxic effect of bile acids and the cholestatic agent ANIT in HPC. A-E.** Cells were serum starved and treated or not for 24 h with: **(A)** different concentrations of GCDC (1-2mM) + TCA (0.5-1mM). Cell viability was analysed by crystal violet staining. Data are mean± S.E.M. of 2-6 experiments run in duplicate; **(B)** different concentrations of GCDC (0.1-2mM) + TCA (0.5mM). Cell viability was analysed by MTT assay. Data are mean ± S.D. from one representative experiment run in sextuplicates; **(C)** different concentrations of TCDC (25-1000 μM). Cell viability was analysed by crystal violet staining. Data are mean± S.E.M. of 3-6 experiments run in duplicate; **(D)** different concentrations of ANIT (1-50μM). Cell viability was analysed by crystal violet staining; **(E)** bile from 3 different mice in different dilutions (1:4; 1:6 and 1:8). Cell viability was analyzed by cell counting with Neubauer Chamber. Data are mean± S.E.M. of 3 experiments run in duplicate.** F-H.** HPC, primary mouse hepatocytes and immortalized mouse hepatocytes were treated with: **(F)** GCDC (1-1.5mM) + TCA (0.5mM); **(G)** TCDC (100 μM); **(H)** ANIT (10-25μM) for 24 h. Cell viability was analysed by crystal violet staining. Data are mean± S.E.M. of 3-8 experiments run in triplicate. **I.** Caspase-3 activity in HPC treated or not with GCDC (1mM) + TCA (0.5mM) or TCDC (100-200μM) for 8h or 16 h. Data are mean ± S.E.M. of 4-6 independent experiments and are expressed as fold change of untreated cells (8h). **J.** LDH release assay in HPC treated or not with GCDC (1mM) + TCA (0.5mM) or TCDC (100-200μM) for 15h or 24 h. Data are mean ± S.E.M. (n=6). **A-J**: Data were compared with the untreated group or as indicated, *p<0.05; **p<0.01 and ***p<0.001.

Bile acids are known to exert a cytotoxic effect in hepatocytes 39. Therefore, we first checked their effect on HPC viability by testing different bile acids at different concentrations, alone or in combination, up to a maximum of 2mM, concentration from which detergent effects are already expected 6. We found that combination of 1mM sodium glycochenodeoxycholate (GCDC) and 0.5mM sodium taurocholate (TCA) moderately although significantly reduced cell number, while concentrations above 1.5mM GCDC resulted in a strong cytotoxic effect (Figure 1A-B and Supplementary Figure S1A). Sodium taurochenodeoxycholate (TCDC) elicited a dose response cytotoxic effect (Figure 1C), reaching a nearly maximal response at 200µM concentration, although cytotoxicity was a bit potentiated at 1mM, whereas TCA alone or sodium chenodeoxycholate (CDC) did not have any impact on HPC viability (Supplementary Figure S1B-C). The cholestatic agent 1-naphthyl isothiocyanate (ANIT) 40 also decreased HPC viability in a dose-dependent manner (Figure 1D). Importantly, a dose-dependent cytotoxic effect was also seen in HPC by treatment with bile extracted from mice submitted to BDL confirming that the bile acid pool produced during cholestatic injury in mice elicits a cytotoxic effect (Figure 1E). We compared the bile acids cytotoxic effect in HPC versus hepatocytes using immortalized mouse hepatocytes and primary mouse adult hepatocytes. Our results indicate that HPC are more resistant than hepatocytes to the cytotoxic effects of GCDC + TCA at lower doses (1mM GCDC + 0.5mM TCA) (Figure 1F and Supplementary Figure S1D). No differences were observed in sensitivity to TCDC or ANIT (Figure 1G-H and Supplementary Figure S1D). Evidence indicate that the type of cell death triggered by bile acids depends on different factors 39. We attempted to clarify the type of cell death induced in HPC by performing apoptosis and necrosis specific assays. Our results show that TCDC, but not GCDC+TCA, treatment led to an increase in caspase-3 activity (Figure 1I) whereas GCDC+TCA induced a clear LDH release supporting a necrotic cell death in HPC (Figure 1J). Necrosis was also seen at higher doses of TCDC (Supplementary Figure S1E-F) revealing a dose-dependent induction of apoptosis and necrosis by TCDC. Collectively, these data indicate that bile acids induce HPC cell death, and both the degree of cell sensitivity and the mechanism of cell death depend on the bile acid species and the concentration. Based on this primary screening analysis, we selected the combination GCDC 1mM + TCA 0.5mM and the treatment with TCDC 100μM alone as representative bile acids for further studies.

**Figure 2:**
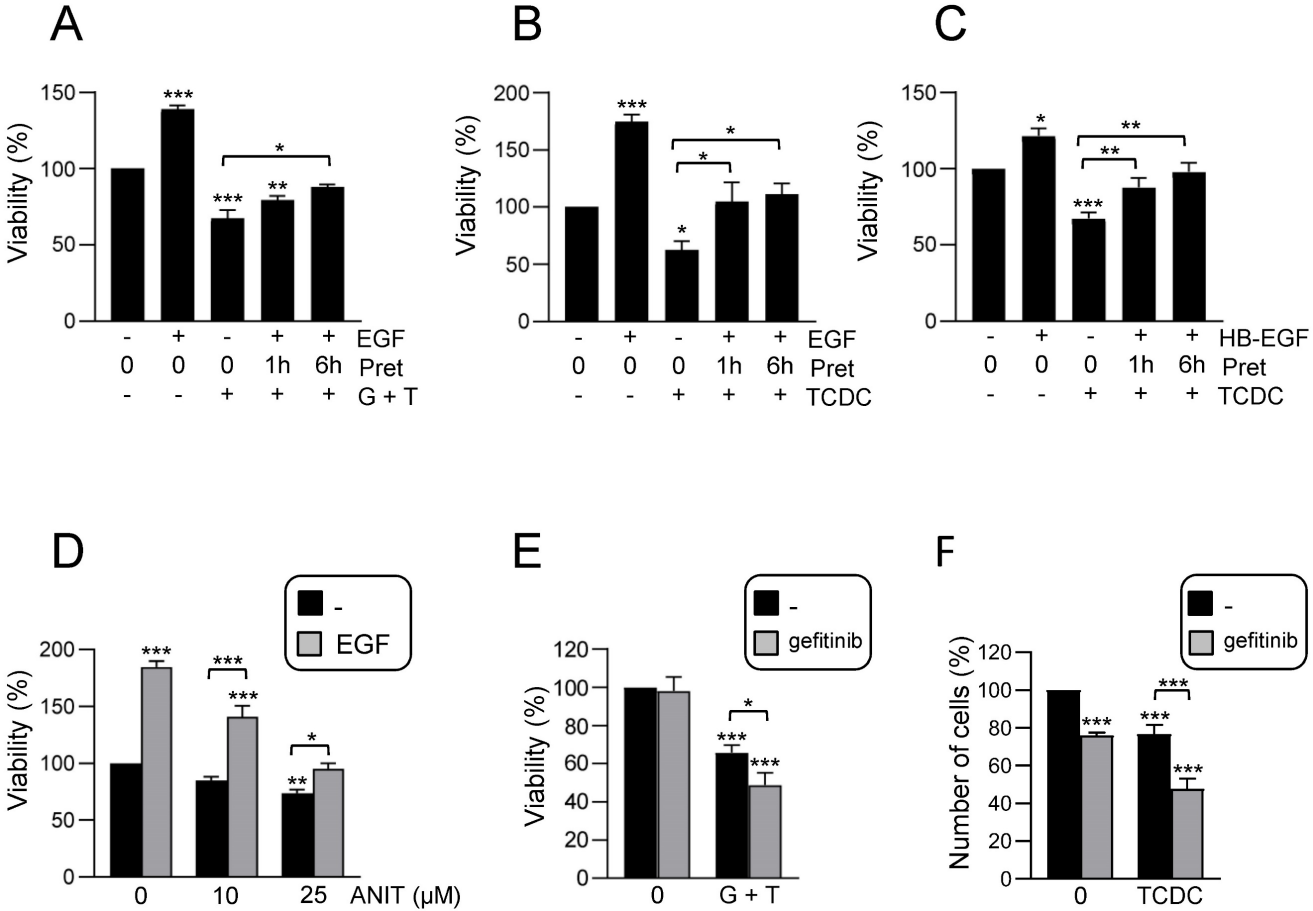
** Effect of EGFR ligands on cell viability in HPC treated with bile acids. A-C.** Cells were serum starved and treated or not for 1 or 6 h with **(A-B)** EGF (20 ng/mL) or **(C)** HB-EGF (20 ng/mL) prior to adding **(A)** GCDC (1mM) + TCA (0.5mM) (G+T); **(B-C)** TCDC (100μM). Cell viability was analysed after 24 h by crystal violet staining. Data are mean± S.E.M. of 3 experiments run in duplicate. **D.** Cells were serum starved and treated or not with ANIT (10 and 25μM) ± EGF (20 ng/mL) (co-treatment). Cell viability was analysed after 24 h by crystal violet staining. Data are mean± S.E.M. of 6 experiments run in duplicate. **E-F.** Cells were serum starved, pretreated for 1 h with gefitinib (2.5μM) and treated for 24 h with **(E)** GCDC (1mM) + TCA (0.5mM) (G+T) or **(F)** TCDC (100μM). Cell viability was analysed by crystal violet staining in **(E)** or by cell counting with Neubauer chamber in **(F)**. Data are mean± S.E.M. of 4 and 3 experiments, run in duplicate or triplicate, respectively. **A-F**. Data were compared with the untreated group or as indicated, *p<0.05; **p<0.01 and ***p<0.001.

As mentioned above, evidence support a key regulatory role for EGFR signaling pathway during cholestatic damage 23. In fact, bile acids activate EGFR in HSC and cholangiocytes leading to either cell growth or apoptosis 41, 42 but no information is available regarding HPC. This prompted us to analyse a potential role for EGFR activity in the cytotoxic response triggered by bile acids in HPC. EGF protected against the loss of cell viability induced by bile acids when added 1-6 h ahead of bile acids treatment (Figure 2A-B and Supplementary Figure S2A-B), but not when ligand was added simultaneously to bile acids (Supplementary Figure S2C). Similar protection was obtained when using HB-EGF, another EGFR ligand, instead of EGF (Figure 2C). EGF also blocked ANIT cytotoxicity (Figure 2D). We confirmed the EGF protective effects on HPC response to bile acids using gefitinib, an EGFR tyrosine kinase inhibitor 43. Efficacy of gefitinib to block EGF-induced signaling was documented by an impairment in EGFR phosphorylation by ligand stimulation (Supplementary Figure S3A) and impediment of EGF-induced biological activity in HPC, specifically its mitogenic activity (Supplementary Figure S3B). Data evidenced that incubation with gefitinib further sensitized HPC to the cytotoxic effect of bile acids (Figure 2E-F), again demonstrating the protective role of EGFR signaling in this context.

### EGFR signaling has a key role in the inflammatory response induced by bile acids in hepatic progenitor cells

It has been described that bile acids can trigger an inflammatory response in hepatocytes as part of their cytotoxic mechanism 9, 10, however if this also occurs in HPC is not known. To explore this possibility, we treated HPC with TCDC or GCDC+TCA and analysed the activation of inflammatory signaling pathways, STAT3, p38MAPK and NF-κB. Results show that death induced by bile acids is accompanied by phosphorylation of p38MAPK and STAT3 in HPC, indicating an activation of these pathways.

**Figure 3:**
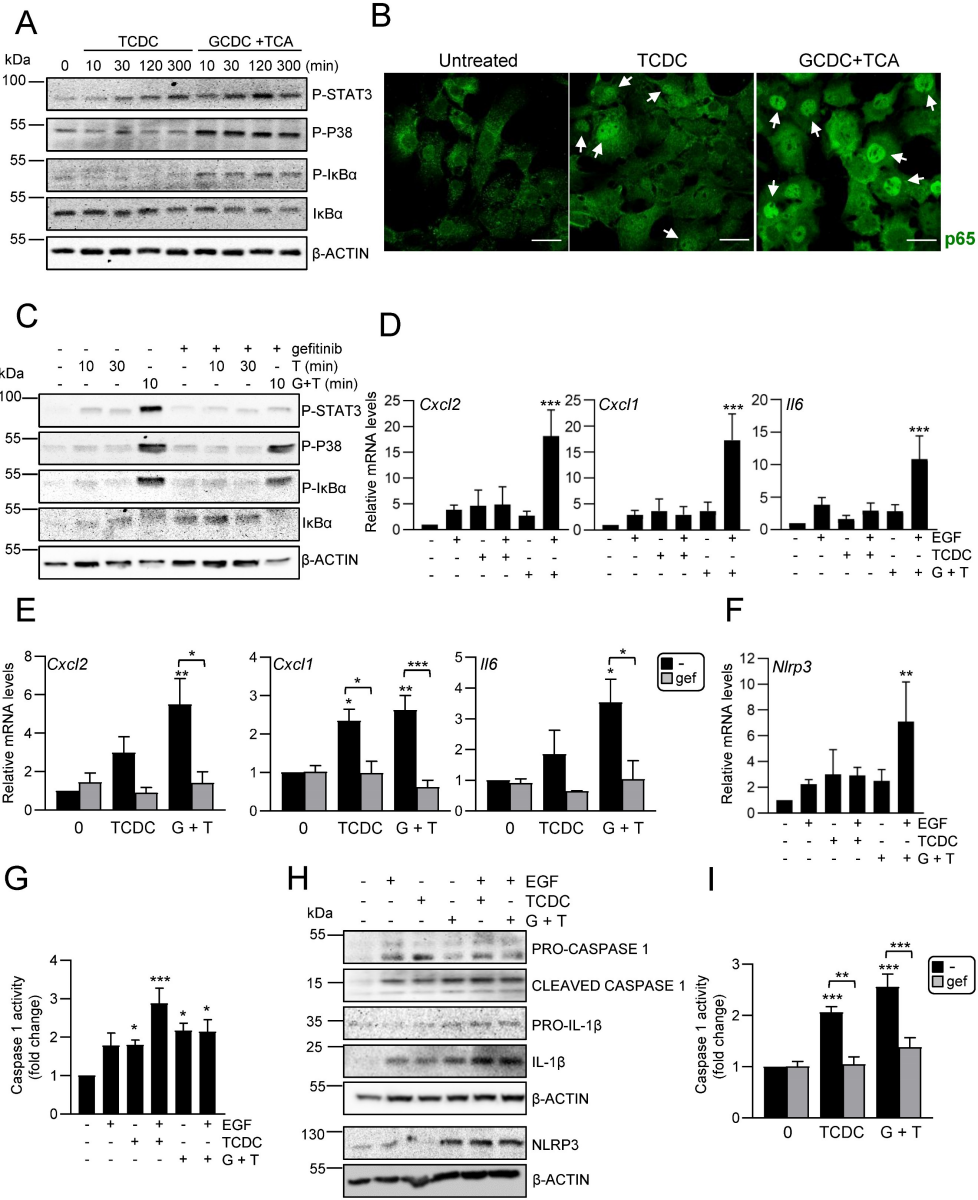
** Activation of an inflammatory response by bile acids in HPC: Involvement of the EGFR pathway. A.** Western blot analysis for phosphorylated STAT3 (P-STAT3), p38MAPK (P-P38) and IκBα (P-IκBα), and total IκBα, in HPC treated or not with TCDC (100μM) or GCDC (1mM) + TCA (0.5mM) for different periods of time. A representative experiment out of 5 is shown. **B.** Confocal microscopy images of p65 staining in cells treated or not with TCDC (100μM) or GCDC (1mM) + TCA (0.5mM) for 30 min. An Alexa 488-conjugated secondary antibody was used. Representative images out of 2 experiments are shown. Scale bar =30 μm. Arrows mark p65 nuclear translocation. **C.** Western blot analysis for phosphorylated STAT3 (P-STAT3), p38MAPK (P-P38) and IκBα (P-IκBα), and for total IκBα in HPC pretreated for 1h with gefitinib (2.5μM) prior to adding TCDC (100μM) (T) or GCDC (1mM) + TCA (0.5mM) (G+T) for 10 or 30 min. Representative blots are shown. **D-E.** RT-qPCR analysis of the expression of *Cxcl2, Cxcl1* and* Il6* in HPC treated or not with: **(D)** EGF (20ng/mL), TCDC (100μM) or GCDC (1mM) + TCA (0.5mM) (G+T) for 15 h (*Cxcl2* and* Cxcl1*) or 1 h (*Il6*); **(E)** gefitinib (2.5μM) for 1 h prior to adding TCDC (100μM) or GCDC (1mM) + TCA (0.5mM) (G+T) for 15 h (*Cxcl2* and* Cxcl1*) or 1 h (*Il6*). *Gusb* was used for normalization. Data are expressed relative to untreated cells (assigned an arbitrary value of 1) and are mean of 3-7 independent experiments. **F.** RT-qPCR analysis of the expression of *Nlrp3* in HPC treated or not with EGF (20ng/mL), TCDC (100μM) or GCDC (1mM) + TCA (0.5mM) (G+T) for 1 h. *Gusb* was used for normalization. Data are expressed relative to untreated cells (assigned an arbitrary value of 1) and are mean of 3-7 independent experiments. **G.** Caspase 1 activity in cells treated or not with EGF (20 ng/mL), TCDC (100μM) or GCDC (1mM) + TCA (0.5mM) for 1 h. Data are mean ± S.E.M. of 5-10 independent experiments and are expressed as fold change of untreated cells (assigned an arbitrary value of 1). **H.** Western blot analysis for PRO-CASPASE 1, CLEAVED-CASPASE 1, PRO-IL-1β, IL-1β and NLRP3 in HPC treated or not with EGF (20 ng/mL), TCDC (100μM) or GCDC (1mM) + TCA (0.5mM) (G+T) for 30 min. A representative experiment out of 3 is shown. **I.** Caspase 1 activity in cells pretreated for 1 h with gefitinib (2.5μM) prior to adding TCDC (100μM) or GCDC (1mM) + TCA (0.5mM) (G+T) for 1 h. Data are mean ± S.E.M. of 4 independent experiments and are expressed as fold change of untreated cells (assigned an arbitrary value of 1). **D, F, G, H.** EGF and bile acids were added simultaneously. **A-I**. Data were compared with the untreated group or as indicated, *p<0.05; **p<0.01 and ***p<0.001.

An increase in phosphorylated IκBα and decrease in IκBα levels was also observed, which together with nuclear translocation of p65, showed the activation of the NF-κB. In all cases, a higher activation is seen with GCDC + TCA compared to that observed when death is induced by TCDC (Figure 3A-B and Supplementary Figure S4A). Furthermore, we checked if bile acids induce the expression of inflammatory mediators in HPC. RT-qPCR analysis of inflammatory cytokines and chemokines genes in HPC treated with GCDC + TCA or TCDC (Supplementary Figure S4B) demonstrated that bile acid treatment increases expression of different interleukins (*Il6, Il1b, Il4*), chemokines (*Cxcl2, Cxcl1, Ccl2*) and the cytokine *Tnfa* in HPC.

Together, these data evidence that bile acids trigger an inflammatory response in HPC. Since our recent work revealed a key role for EGFR promoting the pro-inflammatory response activated during cholestatic injury 23, we next explored whether EGFR signaling is involved in the bile acids-induced inflammatory response in HPC. We found that bile acids trigger EGFR activation as illustrated by an increase in phosphorylated EGFR (Supplementary Figure S4C). Furthermore, EGFR signaling is required for bile acid-induced pro-inflammatory signaling, as incubation with gefitinib interfered with STAT3, p38MAPK and IκB phosphorylation (Figure 3C and Supplementary Figure S4D). Since levels of EGFR ligands are elevated in cholestatic diseases 23, 44, we analysed the effect of bile acids and EGF co-treatment. Importantly, bile acids (particularly GCDC + TCA) and EGF show a synergistic effect up-regulating *Cxcl2, Cxcl1* and *Il6* expression (Figure 3D). Furthermore, pretreatment with the EGFR inhibitor blocked the up-regulation induced by bile acids (Figure 3E) further reinforcing the contribution of EGFR on the bile acid inflammatory response in HPC. To further characterize the molecular features of this inflammatory response, we analysed a potential activation of NLRP3 inflammasome since it is expressed in both parenchymal and non-parenchymal liver cells and its activation has lately gained relevance in the pathogenesis of various types of liver diseases 45 although no information is available in regard to its expression or activation in HPC. An increased expression of *Nlrp3* was seen in HPC treated with TCDC, GCDC+TCA, or EGF, and again its levels were significantly upregulated following co-treatment of GCDC+TCA with EGF (Figure 3F). Similar data were obtained at the protein level (Figure 3H and Supplementary Figure S5A), although statistical significance was not reached here due to variations in the strength of induction. Next, we assessed NLRP3 function by analysing caspase-1 activation and found increased enzymatic activity (Figure 3G) and appearance of the cleaved/active form of caspase-1 in HPC treated with bile acids and EGF, especially under the combined treatment (Figure 3H-I and Supplementary Figure S5A). Once activated, caspase-1 cleaves pro-IL-1β to release IL-1β. Consistently, we found increased IL-1β protein levels upon treatment with bile acids alone or in combination with EGF (Figure 3H and Supplementary Figure S5A). Importantly, the fact that both bile acids-induced *Nlrp3* expression (Supplementary Figure S5B) and caspase-1 activity (Figure 3I) are prevented in the presence of gefitinib demonstrate a role as well for EGFR in NLRP3 inflammasome activation. Overall, we demonstrate that bile acids trigger an inflammatory response in HPC in which the activation of EGFR plays a main role. Besides, exogenous EGF further potentiates bile acid-induced upregulation of inflammatory mediators and NLRP3 inflammasome activation.

### Hepatic progenitor cells´ secretome analysis reveals an immunoregulatory function and a central role in hepatic cell-cell communication

**Figure 4:**
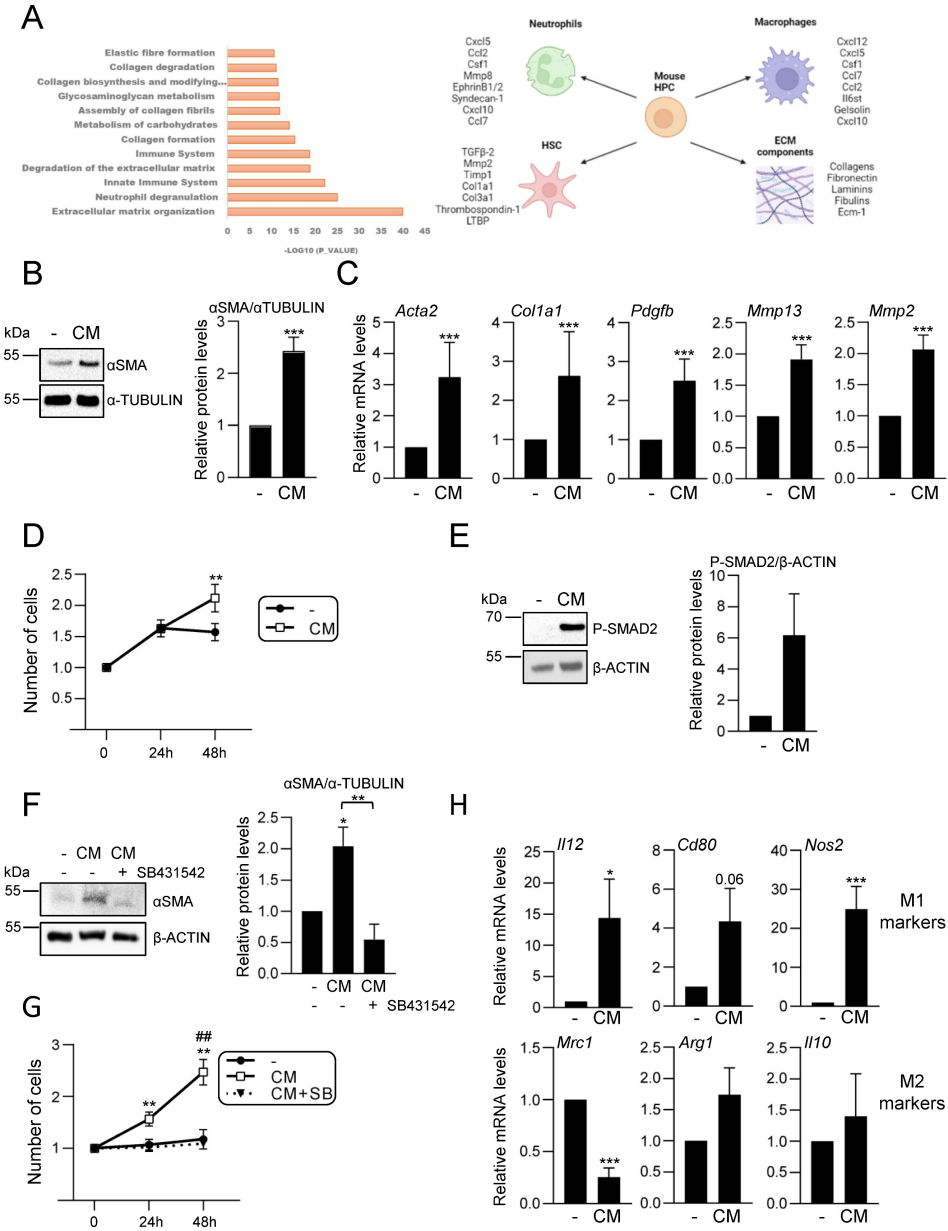
** Analysis of HPC secretome. Effect on HSC and macrophages. A.** Top significantly enriched reactome pathways in HPC secretome using proteomic data and PANTHER overrepresentation test (left panel). ECM components and proteins involved in intercellular communication with neutrophils, macrophages and HSC are indicated (right panel). **B.** Western blot analysis for αSMA in GRX cells after 24 h incubation in the absence (-) or presence (CM) of HPC conditioned medium. A representative experiment is shown (left panel). Optical density values are mean ± S.E.M. of 8 independent experiments (right panel). **C.** RT-qPCR analysis of the expression of HSC activation markers in GRX cells after 8h (*Col1a1, Acta2*,* Pdgfb)* or 24 h (*Mmp13* and* Mmp2*) incubation in the absence (-) or presence (CM) of HPC conditioned medium. *Gusb* was used for normalization. Data are expressed relative to cells in the absence of CM (assigned an arbitrary value of 1) and are mean ± S.E.M. of 4-7 independent experiments. **D.** Cell counting of GRX cells incubated for 24 or 48 h in the absence (-) or presence (CM) of HPC conditioned medium. Data are expressed relative to day 0 and are mean ± S.E.M. of 3 independent experiments run in triplicate. **E.** Western blot analysis for phosphorylated SMAD2 (P-SMAD2) in GRX cells after 30 min incubation in the absence (-) or presence (CM) of HPC conditioned medium. A representative experiment is shown (left panel). Optical density values are mean ± S.E.M. of 4 independent experiments (right panel). **F.** Western blot analysis for αSMA in GRX cells after 24 h incubation in the absence (-) or presence (CM) of HPC conditioned medium with (+) or without (-) 2 h pretreatment with SB 431542 (10 μM). A representative experiment is shown (left panel). Optical density values are mean ± S.E.M. of 3 independent experiments (right panel). **G.** Cell counting of GRX cells incubated for 24 h or 48 h in the absence (-) or presence (CM) of HPC conditioned medium with (+) or without (-) 2 h pretreatment with SB431542 (10μM). Data are expressed relative to day 0 and are mean ± S.E.M. of 3 independent experiments run in triplicate. **H.** RT-qPCR analysis of the expression of M1 (*Il12, Cd80, Nos2*) and M2 markers *(Mrc1, Arg1, Il10)* in mouse peritoneal macrophages incubated for 24 h in the absence (-) or presence (CM) of HPC conditioned medium.* Gusb* was used for normalization. Data are expressed relative to macrophages incubated without CM (assigned an arbitrary value of 1) and are mean ± S.E.M. of 4-6 independent experiments. **A-H**. Data were compared with the cells without CM: *p<0.05; **p<0.01 and ***p<0.001. **G**: ##*p*<0.01: Cells incubated with CM with (CM+SB) versus without (CM) SB pretreatment**.**

Our data support an active role for HPC in the hepatic inflammatory response associated to cholestatic injury. These findings prompted us to analyse in detail the secretome profile of mouse HPC to better understand the role of this cell population in the injured liver. For that, we initially performed a liquid chromatography-tandem mass spectrometry (LC-MS/MS)-based proteomic analysis of the secretome using HPC-derived conditioned medium generated as described in material and methods section. Reactome pathway analysis identified among the top significantly enriched pathways those related to neutrophil and platelet degranulation, and immune system (Figure 4A). Indeed, the top secreted proteins include a number of cytokines and chemokines involved in neutrophil and macrophage regulation (Figure 4A, right panel). Interestingly, other enriched reactome pathways were related to the extracellular matrix (ECM) organization and degradation, including collagen biosynthesis, assembly and degradation, and glycosaminoglycans metabolism. Additionally, several proteins involved in the activation of HSC to myofibroblasts are secreted by HPC. These data evidence that HPC secrete a large number of proteins involved in cell-cell communication, including not only inflammatory mediators but also proteins involved in ECM remodeling, which directly and indirectly contribute to the intercellular dialogue. Based on these interesting findings, we chose to further analyse the postulated communications between HPC and two cell populations with a critical role in liver injury, HSC and macrophages. Incubation of immortalized mouse HSC (GRX cells) with HPC-conditioned medium led to a robust increase of αSMA expression, a marker of activated HSC, both at protein and mRNA level (Figure 4B-C). The expression of other characteristic markers of myofibroblasts, such as *Col1a1, Pdgfb, Mmp13* and* Mmp2*, was also upregulated in HSC treated with HPC-conditioned medium (Figure 4C), confirming that HPC secretome includes factors that trigger HSC activation. Upon activation, HSC show an enhanced proliferation rate, a phenomenon that was also observed in GRX cells treated with HPC-derived conditioned medium (Figure 4D). Since TGF-β2 was identified in HPC secretome, and it has been reported its role in biliary injury and induction of fibrogenic genes in HSC 46, we tested the TGF-β signaling as potential mediator of HSCs activation. An increase in phosphorylated SMAD2 was observed in GRX cells treated with HPC-conditioned medium (Figure 4E), confirming the presence of TGF-β receptor ligands as part of the HPC secretome. More importantly, we demonstrated that secreted TGF-β is required for HSC activation since pre-incubation of GRX cells with SB431542, a TGF-β inhibitor functionally validated in our cellular model (Supplementary Figure S6), impaired both αSMA up-regulation and cell proliferation stimulatory effect induced by HPC-conditioned medium (Figure 4F and 4G). For HPC-macrophage interaction analysis, we tested the effects of HPC-conditioned medium on mouse peritoneal macrophages, specifically, a potential effect on M1- and M2-like phenotype. Interestingly, HPC-conditioned medium induced a significant up-regulation of M1 markers (*Il12, Cd80, Nos2*) while among M2 markers, *Mrc1* was downregulated, and *Arg1* and* Il10* were not modulated (Figure 4H). Altogether these data evidence a relevant role for HPC in the hepatic cellular interactome through the secretion of a plethora of factors that contribute to HSC activation and regulation of macrophage polarity.

### EGFR signaling in combination with bile acids profoundly alters the hepatic progenitor cells´ secretome

**Figure 5:**
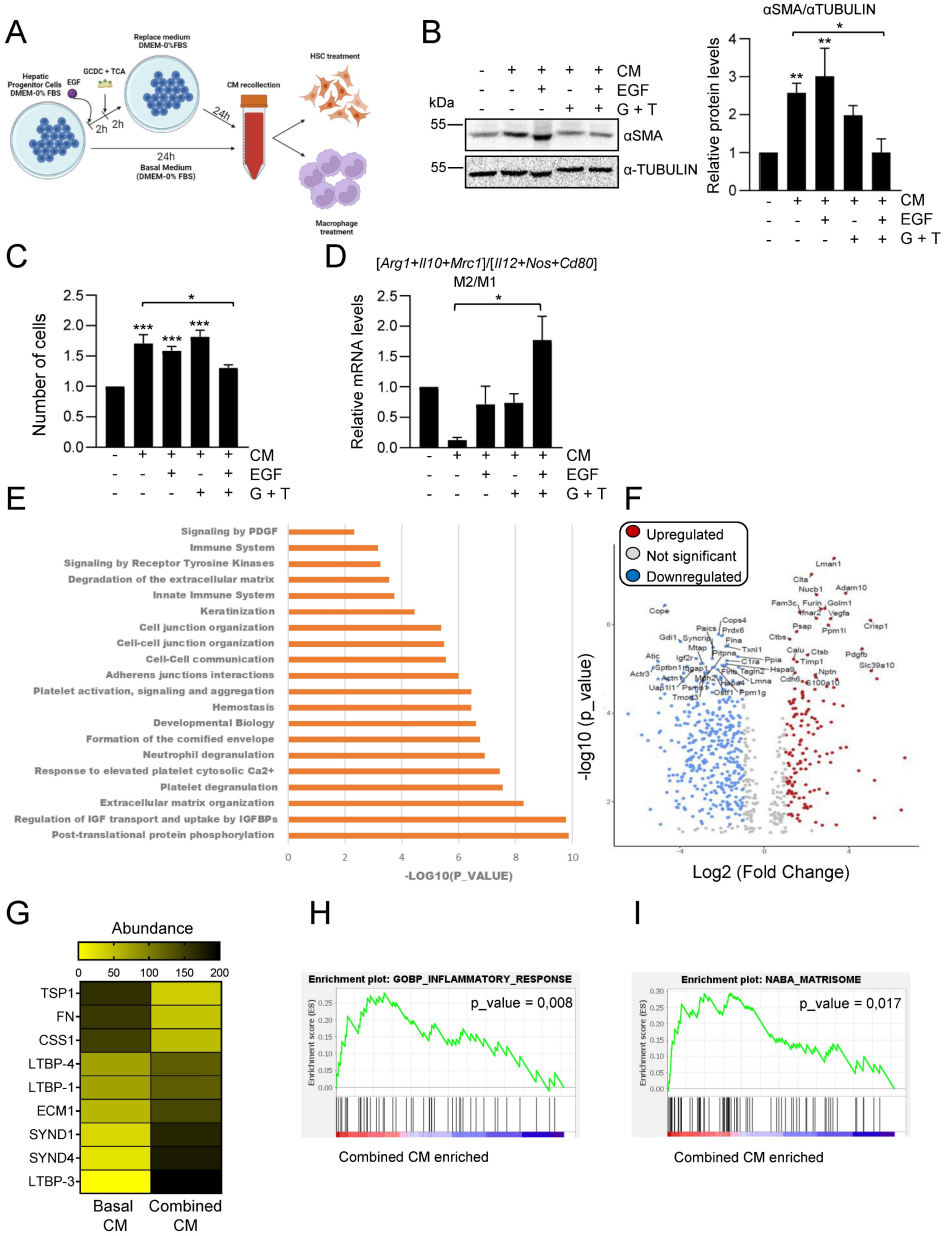
** Changes in HPC secretome by treatment with EGF and bile acids.** Scheme illustrating the experimental procedure applied to assess HPC-conditioned media under different culture conditions. **B.** Western blot analysis for αSMA in GRX cells after 24 h incubation with or without HPC conditioned medium (-/+ CM) generated after incubation in the absence (-) or presence of GCDC (1mM) + TCA (0.5mM) (G+T) and/or EGF (20ng/mL) as indicated in A. A representative experiment is shown (left panel). Optical density values are mean ± S.E.M. of 4 independent experiments (right panel). Data are expressed relative to GRX without CM. **C.** Cell counting of GRX cells treated for 48 h with HPC conditioned media (CM) generated as indicated in A-B. Data are mean ± S.E.M. of 3 independent experiments run in triplicate and are expressed relative to day 0 and GRX without CM. **D.** RT-qPCR analysis of the expression of M1 (*Il12, Cd80, Nos2*) and M2 markers *(Mrc1, Arg1, Il10)* in mouse peritoneal macrophages treated for 24 h with HPC conditioned media (CM) generated as indicated in A-B*. Gusb* was used for normalization. M1 and M2 markers data are analysed as a group. Data are mean ± S.E.M. of 6 different experiments and expressed relative to macrophages without CM. **A-D**. Data were compared with cells without CM or as indicated: *p<0.05; **p<0.01 and ***p<0.001. **E.** Top significantly enriched reactome pathways identified in HPC treated with GCDC + TCA and EGF (combined CM) secretome using proteomic data and PANTHER overrepresentation test. **F.** Volcano plot of comparative proteomics of HPC basal conditioned medium (basal CM) and HPC conditioned medium after treatment with GCDC + TCA and EGF (combined CM). Proteins significantly upregulated (in red, FC>2) or downregulated (in blue, FC<0.5) in the secretome of combined CM are indicated. Top 50 proteins with lower *p* value are labeled. Shift in proteins relative abundance is considered significant if *p* value < 0.05. **G.** Heatmap representing TGF-β processing and bioavailability regulation-related proteins. **H-I**. Gene set enrichment analysis. Y-axis represents enrichment score (ES). X-axis: each black line represents a gene represented in the gene set. Significance threshold set at* p* value <0.05.

Considering our data showing that EGFR signaling has a key modulatory role on the bile acid-induced inflammatory response in HPC (Figure 3), we next investigated whether bile acids or/and EGF changed HPC secretome in any way, by setting up the experimental approach depicted in Figure 5A. Indeed, conditioned medium from HPC treated with combination of EGF and bile acids failed to increase αSMA levels and proliferation in GRX cells (Figures 5B-C). When we compared the effect of different types of HPC-derived conditioned medium on macrophage phenotype, we also found a differential response with conditioned medium from combined treatment, which drives a M2-type response in sharp contrast to the M1 response induced by conditioned medium from untreated HPC (Figure 5D).

These data evidenced a profound alteration in HPC secretome-triggered effects in the presence of bile acids and EGF. To better characterize the impact of bile acids and/or EGF treatment on HPC secretome, we perform a proteomic analysis in CM from untreated HPC (basal CM); HPC treated with EGF (EGF CM); HPC treated with bile acids GCDC+TCA (BA CM) and HPC treated with bile acids GCDC+TCA in the presence of EGF (combined CM). To compare the relative abundance of secretome components, first, the total protein abundance was normalized (Supplementary Figure S7A). Interestingly, principal component analysis (PCA) showed three distinct clusters: the basal secretome, the combined secretome, and the BA and EGF secretomes that grouped together (Supplementary Figure S7B). When we analyzed the significantly enriched reactome pathways in the combined CM *versus* basal CM, we found pathways related to inflammation (neutrophil degranulation, immune system), ECM organization and degradation, and importantly, pathways related to cell-cell junction and communication (Figure 5E). Similarly, immunomodulatory pathways, together with ECM organization and ECM-cell interaction were significantly enriched in combined CM *versus* BA CM or EGF CM (Supplementary Figure S7C-D), but not in BA CM or EGF CM *versus* basal CM (Supplementary Figure S7E-F). These results reinforce the idea of a differential secretome composition in HPC co-treated with bile acids and EGF that reflects an important role in inflammatory/immunomodulatory responses. Therefore, we decided to focus on the comparison between basal CM and combined CM for a more detailed analysis. Among all the differentially secreted proteins (p-value < 0,05) 213 proteins were exclusive of basal CM and 29 exclusive of combined CM and 663 were shared by the two types of conditioned medium (Supplementary Figure S8A), being 179 up-regulated proteins (fold change > 2) and 454 downregulated proteins (fold change < 0.5) in the secretome of combined CM compared to basal CM (Figure 5F). Based on data demonstrating a role for TGF-β pathway in HPC conditioned medium-driven GRX cell activation (Figure 4E-F) we searched for changes linked to the TGF-β pathway that could explain the observed differences (Figure 5B-C) in HSC activation in basal versus combined HPC conditioned medium. No changes were found in levels of TGF-β itself, whereas we observed alteration in the abundance of several proteins implicated in pro-TGF-β processing and regulation of TGF-β bioavailability (e.g. THROMBOSPONDIN 1 (TSP-1), FIBRONECTIN (FN), LATENT TRANSFORMING GROWTH FACTOR BETA BINDING PROTEIN (LTBPs)) (Figure 5G), which could lead to a net decrease on the release and availability of bioactive TGF-β. On the other hand, gene set enrichment analysis revealed important changes in inflammatory response signature (Figure 5H, Supplementary Figure S8B and Supplementary Table S3) with altered levels of immunoregulatory proteins in combined CM, showing enrichment in inflammatory cell-recruiting factors (CXCL5, OSTEOPONTIN or TIMP-1), factors that induce M2-type (COLONY STIMULATING FACTOR 1 (CSF1), GALECTIN-1 (GAL-1)**)** or reduce M1-type (CLUSTERIN) macrophage polarization. Lastly, it is worth highlighting changes found in matrisome gene signature (Figure 5I, Supplementary Figure S8B and Supplementary Table S3). Abundance of pro-fibrotic ECM components (COLLAGEN α-1(III) CHAIN, COLLAGEN α-1(II) CHAIN, FN, FIBULIN) is diminished, while anti-fibrotic markers (EXTRACELLULAR MATRIX PROTEIN-1 (ECM1), SYNDECAN1 and 4 (SDC1, SDC4)) are upregulated in combined CM. Altogether, our data indicate profound changes in HPC secretome upon combined treatment with bile acids and EGF, with a potential direct impact on the outcome of HPC communication with HSC and macrophages.

## Discussion

In this work, we have addressed the HPC response to bile acids as key components of the pathophysiological mechanisms in cholestatic disease. An *in vitro* model of cholestasis was established based on HPC exposure to bile acids at a dose-range emulating the pathological context of cholestasis 3. Particularly, TCA, TCDC and GCDC were chosen as they are the most common bile acids in human bile and serum in cholestasis 11 being TCDC also abundant in mouse bile 47. Our data show that bile acids induce cytotoxic effects on HPC, although outcomes depend on the bile acid species and concentration.

This agrees with previous reports 48, 49 demonstrating varying cytoxicity degree and dose-ranging in hepatocytes that may depend upon the specific hydrophobicity and conjugation status of bile acids, among other potential factors. Strikingly, TCA was reported to induce cell proliferation and biliary differentiation in PIL2 murine liver progenitor cell line 50. In our hands, TCA by itself failed to induce a cytotoxic response in HPC and no signs of cell proliferation were detected. This apparently discrepant results might be explained by the tumorigenic nature of PIL2 cells, which are p53 null cells 51, therefore likely influencing their response. Our data also suggest that at least in certain cases HPC might have advantage over hepatocytes in terms of resistance to bile acid injury, an interesting and potentially relevant issue that nonetheless requires further research. The cell death mechanisms of bile acids in hepatocytes depend as well on the specific bile acid and cell type, and both apoptosis and necrosis have been reported. As an example, GCDC triggers apoptosis in human hepatocytes, whereas necrosis in mouse and rat hepatocytes *in vitro* 11. We show that TCDC treatment in HPC results in increased caspase-3 and caspase-1 activities suggesting the induction of both apoptosis and pyroptosis 52. No caspase-3 activity was apparently induced by GCDC+TCA, but appearance of an annexin V(+)/propidium iodide(-) cell population was detected (data not shown), which would support apoptotic cell death, concomitantly with necrotic cell death evidenced by the increase in LDH release. These data reflect the complex nature of the cell death response triggered by bile acids in HPC, while support the possible coexistence of various cell death processes, an issue that also deserves future investigation. Independently of the type of cell death, EGFR ligands impair bile acid cytotoxicity, in line with results observed in hepatocytes using Amphiregulin 44. Intriguingly, this effect seems specific of EGFR as HGF, another receptor tyrosine kinase (RTK) ligand, does not elicit cytoprotection (data not shown). Besides, the intricate crosstalk between EGFR and bile acids goes beyond the protective effect of EGFR ligands, as both GCDC+TCA and TCDC activate the EGFR pathway. Activation of EGFR by bile acids has been observed in other liver cells, through both ligand dependent 42 and independent mechanisms 41, 53, 54. Nonetheless, the functional outcome of the bile acid-EGFR signaling axis crosstalk is again unclear. Our data in HPC agrees with the cytoprotective effect of EGFR activation seen in hepatocytes 53, but others have shown an implication in bile acid-induced apoptosis also in hepatocytes 54, while in HSC it can drive proliferation and apoptosis 41. These results indicate that distinct intrinsic and extrinsic factors likely constrain the final effect according to the cellular, signaling, and molecular context.

Beyond bile acid cytotoxicity, in recent years it has become clear that liver injury is fueled by the cooperation of different processes, including inflammation and bile acid actions as signaling molecules. Here, we show that HPC are key players in the activation and regulation of the inflammatory response associated to cholestatic liver injury. The bile acid-induced chemokines and cytokines expression in HPC is in line with previous data in hepatocytes demonstrating its contribution to a pro-inflammatory environment 9, 55. Although the interaction between bile acids and EGFR is well established, the implication of EGFR activation in bile acid-mediated inflammatory signaling is not so well defined. Certainly, EGFR activation by bile acid has been shown to drive JNK activation and COX2 induction in cholangiocarcinoma cells 56 evidencing a functional connection in a liver tumour context. Now, we prove that the bile acid-EGFR-inflammation interacting axis could operate as well in cholestatic injury, a non-tumour context, having HPC as cellular mediator. Thus, we demonstrate that bile acids trigger, via EGFR, the activation of inflammation-related pathways in HPC; and bile acids and EGF synergistically increase cytokines expression, while promoting NLRP3 inflammasome activation. The activation of NLRP3 is particularly interesting. Although its activation and implication in cholestatic injury is recognized, its specific role and circuit of action is not fully clear. Some evidences fully support a critical role for pyroptotic cell death by NLRP3 in aggravation of biliary injury 57, 58, whereas others rather defend that it is not directly activated by bile acids in hepatocytes nor macrophages, and its absence ameliorates liver injury to some degree but increases fibrosis 59, 60 offering an interesting and challenging scenario. We do not see a correlation between cytotoxicity and inflammation in HPC, since combination of EGF and bile acids reduces cell death but potentiates inflammation and NLRP3 activation, providing additional support for a versatile and tuneable role for the inflammasome in cholestasis that awaits further characterization.

We also provide here novel evidence on the HPC actions in the dynamic and complex cell-cell dialogue established during the regenerative response of the injured liver. Previous studies in adult-derived human liver stem/progenitor cells already indicated that HPC have the ability to secrete a number of cytokines and other mediators involved in inflammation, suggesting immunomodulatory effects 61, 62. Our proteomic analysis of mouse HPC secretome strengthen these observations. In fact, mouse HPC do secrete many proteins involved in intercellular communication with neutrophils, macrophages, and HSC, as well as proteins involved in ECM remodeling, which, apart from its evident relevance on HPC migratory activity (an absolute requirement for their regenerative potential), is known to directly impact on release and bioavailability of growth factors, thus adjusting intercellular communication 63. Regarding HPC-HSC communication, their local proximity and tight interaction during liver regeneration is known, but the established idea is that HSC regulate HPC proliferation and activation through secreted factors (Lymphotoxin-beta, IL-6, or growth factors, including HGF and FGFs, among others) 64, 65. Our data and others evidence 61, 62 that HPC synthetize and release factors capable of modulating HSC. An inhibitory effect on HSC activation was reported for adult-derived human stem/progenitor cells 66. However, in the present study we clearly show that HPC-derived CM activates HSC based on αSMA and other myofibroblast markers expression, and increased cell proliferation, effects that are TGF-β-dependent. We also prove that HPC can modulate macrophage phenotype. A wide macrophage molecular and functional diversity has been recently described by single cell technologies 67. Nonetheless, the simplified classic division into M1 (pro-inflammatory) and M2 (anti-inflammatory/pro-restorative) subtypes provides useful information. Previous reports have demonstrated that macrophages regulate HPC 68, 69. Our findings highlight a more complex crosstalk by showing that HPC regulate macrophage polarization promoting an M1-like phenotype, thus uncovering a bidirectional cellular regulatory action. Additional work is necessary to identify the specific cytokine/s responsible for this effect, but for now proteomics analysis provides a few candidates, such as CCL2 and CXCL10, drivers of M1 macrophage polarization 70, 71.

Overall, our data support the notion that HPC secretome can drive HSC activation and M1-like macrophage polarization, thus contributing to create a pro-inflammatory and pro-fibrotic hepatic microenvironment in the injured liver. Strikingly, upon treatment with bile acids and EGF HPC secretome drastically changes, and subsequently also the biological effects on HSC and macrophages, being the effect on HSC activation completely blunted and switching macrophage polarization to M2-like phenotype. These findings highlight the dynamic and adjustable nature of HPC action, well consistent with the known dual role these cells play during liver damage 17. The mechanisms behind these changing responses await further characterization. Since we show that TGF-β is responsible for HPC basal CM-driven HSC activation, we hypothesized that lack of HSC activation by HPC treated CM could rely on alterations in activation of TGF-β pathway. We found indirect evidence pointing in this direction. Precisely, HPC treated CM shows lower relative abundance of FN, implicated in large latent TGF-β complex assembly and deposition into the ECM 72, and a higher abundance of ECM1, a novel regulator with an inhibitory effect on latent TGF-β activation 73. TSP1 and CALPAIN 1 (CSS1), proteases that participate in the activation of TGF-β 73, are also decreased, which altogether support changes in the matrisome likely affecting TGF-β bioavailability.

Likewise, important changes were observed in the secretome of treated HPC in relation with inflammation, with an enrichment in inflammatory cell-recruiting factors (CXCL5, SECRETED PHOSPHOPROTEIN 1 (SPP1) or TIMP1), macrophage M2-inducing factors (CSF1, GALECTIN 1 (LGALS1), CCN4, CXCL16) or factors interfering with M1-like macrophage polarization (CLU), which could well be behind the effect observed on macrophages. It is also worth mentioning the presence of neutrophils recruitment and degranulation factors (ADAM10, TIMP1, CXCL5). Neutrophil recruitment is part of the inflammatory response to pathogens or injury signals, and although generally associated to hepatocyte toxicity in early stages of the cholestatic injury, they have been also associated with repair, and an indispensable role in tissue regeneration has been granted 74, 75. An in-depth analysis of HPC and neutrophils communication is pending and will help to clarify the complex network of interactions established between HPC and immune cells in the context of cholestasis.

In summary, this work contributes to clarify HPC response under a cholestatic damage. Notably, we have elucidated a novel contribution of HPC in the pathophysiology of the cholestatic liver disease. Far beyond their capability to repopulate the liver by giving rise to hepatocytes and cholangiocytes and thus compensating cell loss during liver damage, we evidence that HPC, through the secretion of growth factors, cytokines, chemokines and ECM assembly and remodeling proteins, contribute to remodel the microenvironment relevantly impacting on the regenerative process by directly modulating other hepatic cells phenotype and functional properties. Our data further support HPC plasticity, specifically in terms of their secretome, consistent with their differential fate in different pathological contexts and microenviroments. We also uncover a key role for EGF on regulation of HPC response during cholestatic damage that might be determinant for the outcome of the cholestatic injury.

## Supplementary Material

Supplementary figures and tables.
