# Supplementary material for: Uncovering a Novel Functional Interaction Between Adult Hepatic Progenitor Cells, Inflammation and EGFR Signaling During Bile Acids-Induced Injury
Source: Int J Biol Sci. 2024 Apr 8;20(7):2339–55. doi: 10.7150/ijbs.90645 (PMC11077361; doi:10.7150/ijbs.90645)

SUPPLEMENTARY INFORMATION

Supplementary figures legends

Supplementary figure 1. Cytotoxic effect of single bile acids in HPC.

A-C. Cells were serum starved and treated or not for 24h with different concentrations of **(A)** GCDC (0.1 -2mM); **(B)** TCA (0.1-2mM); **(C)** CDC (25-200Mm). In all cases, cell viability was analyzed by crystal violet staining. **D.** HPC and primary mouse hepatocytes were treated or not with GCDC (1mM) + TCA (0.5mM) or TCDC (10 µM) for 24h. Cell viability was analyzed by cell counting with Neubauer chamber. **A-D**. Data are mean ± SEM of 3-5 independent experiments run in duplicate. Data were compared with the untreated group or as indicated, **p*<0.05 and ****p*<0.001.

Supplementary figure 2. Effect of EGFR ligands on cell viability in HPC treated with bile acids.

A-B. Cells were serum starved and treated or not for 1 or 6 h with EGF (20 ng/ml) prior to adding **(A)** GCDC (1mM) + TCA (0.5mM) (G+T) or **(B)** TCDC (100µM). Cell viability was analyzed after 24h by cell counting with Neubauer chamber. Data are mean ± SEM of 4 independent experiments run in duplicate. **C**. Cells were serum starved and treated or not with TCDC (100µM); GCDC (1mM) + TCA (0.5mM) or GCDC (1.5mM) + TCA (0.5mM) ± EGF (20 ng/ml). Cell viability was analyzed after 24h by crystal violet staining. Data are mean ± SEM of 3-6 independent experiments run in duplicate. **A-C**. Data were compared with the untreated group or as indicated, **p*<0.05; ***p*<0.01 and ****p*<0.001.

Supplementary Figure 3. Validation of the EGFR inhibitory activity of gefitinib in HPC.

A. Western blot analysis for phosphorylated and total EGFR in HPC treated for 1h with different concentrations of gefitinib (1-5µM) prior to adding EGF (20 ng/ml) for 10 minutes. A representative experiment out of 2 is shown. Optical density values are mean± SEM of 2 independent experiments. **B.** Cells were serum starved, treated for 1h with different concentration of gefitinib (2.5-5µM) prior to adding EGF (20 ng/ml). Cell viability was analyzed after 24h by crystal violet staining. Data are mean ± SD of 1 experiment run in triplicate. Data were compared with the untreated group or as indicated, **p*<0.05; ***p*<0.01 and ****p*<0.001.

Supplementary Figure 4. Analysis of inflammatory pathways in HPC treated with bile acids and/or EGF.

A. Optical density values from western blot analysis (shown in main figure 3A) for P-STAT3, P-P38MAPK and P-IκBα/IκBα in HPC treated with TCDC (100µM) or GCDC (1mM) + TCA (0.5mM) for the indicated periods of time. Data are mean ± S.E.M. of 5 independent experiments.

B. RT-qPCR analysis of the expression of *Cxcl2, Cxcl1, Il6, Tnfa, Il1b, Il4* and *Ccl2* in HPC treated with TCDC (100µM) or GCDC (1mM) + TCA (0.5mM) for 15h. *Gusb* was used for normalization. Data are expressed as fold change relative to untreated cells (assigned an arbitrary value of 1) (n=2). **C**. Western blot analysis for phosphorylated (P-EGFR) and total EGFR in HPC treated with TCDC (100µM) or GCDC (1mM) + TCA (0.5mM) for different periods of time. A representative experiment is shown. Optical density values are shown below the blots´ images and are mean± SEM of 4 independent experiments. **D.** Optical density values from western blot analysis (shown in main figure 3G) for cleaved CASPASE 1 and IL-1β of HPC treated with EGF (20 ng/ml) and/or TCDC (100µM) or GCDC (1mM) + TCA (0.5mM) (G+T) for 30 min. Data are mean ± S.E.M. of 3 independent experiments. **A, C, D**: Data were compared with the untreated group, *$p<0.05$ and **$p<0.01$.

Supplementary Figure 5. Validation of the TGF-β receptor inhibitory activity of SB431542 in HPC.

Western blot analysis for phosphorylated SMAD2 (pSMAD2) in HSC treated for 30 minutes with TGF-β (2ng/ml) with (+) or without (-) 1h pretreatment with SB431542 (10 µM). A representative experiment is shown. Optical density values are shown below the blots´ images.

Supplementary Figure 6. Proteomic analysis of conditioned medium from HPC either untreated or treated with bile acid and EGF.

A. Representation of total protein abundance in conditioned medium from untreated HPC (basal CM) or from HPC treated with GCDC + TCA and EGF (treated CM), after normalization (n=4). **B.** Principal component analysis of HPC basal and treated CM (n=4) samples from differentially expressed proteins (*p* value<0.05). **C.** Venn diagram showing distribution of differentially secreted proteins in HPC basal and treated CM. **D.** Heatmaps of differentially expressed elements in HPC basal and treated CM within the inflammatory response and matrisome signature. Blue means less relative abundance and red means more relative abundance.

Supplementary table 1. Primer sequences used in quantitative reverse transcriptase-polymerase chain reaction (RT-qPCR).

Protein	Gene	Forward primer (5’-3’)	Reverse primer (5’-3’)
CXCL1	*Cxcl1*	GGTGTCCCCAAGTAACGGAG	AGTGTTGTCAGAAGCCAGCG
αSMA	*Acta2*	ACAGCCCTCGCACCCA	GCCACCGATCCAGACAGAGT

COL1α1	*Col1a1*	GTCGCTTCACCTACAGCACCCT	AGGCGGGAGGTCTTGGTGGTTT
PDGFb	*Pdgfb*	ACTTGAACATGACCCGAGCA	ATCTGGAACACCTCTGTGCG
MMP2	*Mmp2*	CACACCAGGTGAAGGATGTG	GTTGAAGGAAACGAGCGAAG
MMP13	*Mmp13*	TTGGTCCCTGCCCCTTCCCTAT	CGCAAGAGTCGCAGGATGGT
IL6	*Il6*	AGACAAAGCCAGAGTCCTTCA	GGAGAGCATTGGAAATTGGGG
CXCL2	*Cxcl2*	CCACCAACCACCAGGCTACA	TGGCAGGGTCTTCAGGCATT
TNFα	*Tnfa*	ACGTCGTAGCAAACCACCAA	ATCGGCTGGCACCACTAGTT
IL1β	*Il1b*	CTGGGAAACAACAGTGGTCA	CTGCTCATTCACGAAAAGGG
IL4	*Il4*	CTCGAATGTACCAGGAGCCATAT	CACATCCATCTCCGTGCATGG
CCL2	*Ccl2*	CACATCCATCTCCGTGCATGG	ATGTCCTCCTGCACCTCCTC
IL12	*Il12*	TTGAACTGGCGTTGGAAGCA	CGGGTCTGGTTTGATGATGT
Cd80	*Cd80*	ACGTGTCAGAGGACTTCACC	AATCCTGCCCCAAAGAGCAC
NOS2	*Nos2*	AGACCTCAACACAGAGCCCTC	TCGAAGGTGAGCTGAACGAG
MRC1	*Mrc1*	CTAACTGGGGTGCTGACGAG	AGGAGGTTCAGTAGCAGGGA
Arg1	*Arg1*	TAGACAAGCTGGGGGATTGGC	CAAAGCTCAGGTGAATCGGC
IL10	*Il10*	CCTTCAGCCAGGTGAAGACT	GGCAACCCAAGTAACCCTTA
GUSB	*Gusb*	AAAATGGAGTGCGTGTTGGGTCG	CCACAGTCCGTCCAGCGCCTT
NLRP3	*Nlrp3*	TCAGATTGCTGTGTGTGGGA	AGATCCTGACAACACGCGGA

Supplementary table 2. Antibodies used for western blot analysis (CST: Cell Signaling Technology)

**Antibody target**	**Specie**	**Dilution**	**Reference**
P-EGFR	Rabbit	1: 1000	CST 1673777S
EGFR	Goat	1: 100	CST 4267S
β-ACTIN	Mouse	1: 5000	CST 1673700S
α-TUBULIN	Mouse	1:5000	CST 2144S
P-STAT3	Rabbit	1: 1000	CST 1679131S
P-P38	Rabbit	1: 1000	CST 1679211S

P-Iκbα	Rabbit	1: 1000	CST 16772859S
Iκbα	Rabbit	1: 1000	CST 1679242S
αSMA	Mouse	1: 1000	CST A2647
P-SMAD2	Rabbit	1: 1000	CST 3108S
SMAD2	Rabbit	1:1000	CST 5678S
CASPASE-1	Rabbit	1:1000	AdipoGen AG-20B0042-C100
Il-1β	Rabbit	1:1000	Abcam Ab9722

Supplementary table 3. Top 100 proteins significantly upregulated (fold change>2) or downregulated (fold change<0.5) ordered according to their *p* value, in the secretome of HPC conditioned medium after treatment with GCDC + TCA and EGF (treated CM) as compared with HPC basal conditioned medium (basal CM).

Gen Name	Protein Name	Fold Change	P_value
Lman1	Protein ERGIC-53	9,838	3,16E-08
Clta	Clathrin light chain A	4,679	7,27E-08
Adam10	Disintegrin and metalloproteinase domain-containing protein 10	14,282	1,96E-07
Nucb1	Nucleobindin-1	5,538	2,12E-07
Cope	Coatomer subunit epsilon	0,038	3,63E-07
Golm1	Golgi membrane protein 1	7,252	4,31E-07
Furin	Furin	6,391	4,42E-07
Fam3c	Protein FAM3C	3,172	5,28E-07
Ifnar2	Interferon alpha/beta receptor 2	5,506	7,24E-07
Vegfa	Vascular endothelial growth factor A, long form	8,765	7,35E-07
Crisp1	Cysteine-rich secretory protein 1	32,205	7,96E-07
Ppm1l	Protein phosphatase 1L	8,097	1,02E-06
Psap	Prosaposin	2,874	1,44E-06

Gen Name	Protein Name	Fold Change	P_value
Ube2i	SUMO-conjugating enzyme UBC9	0,067	1,61E-05
Sephs1	Selenide, water dikinase 1	0,04	1,65E-05
Cxcl5	C-X-C motif chemokine 5	39,839	1,67E-05
Cct5	T-complex protein 1 subunit epsilon	0,031	1,69E-05
Atp6ap1	V-type proton ATPase subunit S1	10,696	1,73E-05
P4hb	Protein disulfide-isomerase	0,235	1,73E-05
Psme1	Proteasome activator complex subunit 1	0,043	1,78E-05
Lsm2	U6 snRNA-associated Sm-like protein LSm2	0,356	1,82E-05
Rplp0	Large ribosomal subunit protein uL10	0,11	1,87E-05
Hnrnpa1	Heterogeneous nuclear ribonucleoprotein A1	0,135	1,90E-05
Basp1	Brain acid soluble protein 1	3,471	1,92E-05
Uba1	Ubiquitin-like modifier-activating enzyme 1	0,07	1,92E-05
Dpp7	Dipeptidyl peptidase 2	0,454	2,00E-05

Cops4	COP9 signalosome complex subunit 4	0,224	1,66E-06
Prdx6	Peroxiredoxin-6	0,249	1,8E-06
Ctbs	Di-N-acetylchitobiase	2,305	2,13E-06
Gdi1	Rab GDP dissociation inhibitor alpha	0,064	2,43E-06
Flna	Filamin-A	0,298	2,96E-06
Txnl1	Thioredoxin-like protein 1	0,284	3,08E-06
Paics	Phosphoribosylaminoimidazole succinocarboxamide synthetase	0,187	3,28E-06
Pdgfb	Platelet-derived growth factor subunit B	24,65	3,51E-06
Ctsb	Cathepsin B	4,124	4,85E-06
Ppia	Peptidyl-prolyl cis-trans isomerase A	0,414	5,26E-06
Syncrip	Heterogeneous nuclear ribonucleoprotein Q	0,182	5,85E-06
Igf2r	Cation-independent mannose-6-phosphate receptor	0,108	5,87E-06
Calu	Calumenin	2,595	6,08E-06
C1ra	Complement C1r-A subcomponent	0,291	6,5E-06
Atic	Bifunctional purine biosynthesis protein ATIC	0,031	6,85E-06
Timp1	Metalloproteinase inhibitor 1	2,897	6,93E-06
Mtap	S-methyl-5'-thioadenosine phosphorylase	0,133	7,55E-06
Iqgap1	Ras GTPase-activating-like protein IQGAP1	0,125	7,59E-06
Hspa9	Stress-70 protein, mitochondrial	0,289	7,79E-06
Flnb	Filamin-B	0,25	8,31E-06
Lmna	Prelamin-A/C	0,271	8,47E-06
Sptbn1	Spectrin beta chain, non-erythrocytic 1	0,08	8,68E-06
Pitpna	Phosphatidylinositol transfer protein alpha isoform	0,192	9,19E-06
Tagln2	Transgelin-2	0,429	9,2E-06

Cst3	Cystatin-C	3,993	2,06E-05
Vps29	Vacuolar protein sorting-associated protein 29	0,155	2,08E-05
Polr2h	DNA-directed RNA polymerases I, II, and III subunit RPABC3	0,189	2,21E-05
Vcl	Vinculin	0,035	2,27E-05
Dctn2	Dynactin subunit 2	0,287	2,28E-05
Ccl2	C-C motif chemokine 2	7,749	2,51E-05
Scly	Selenocysteine lyase	0,104	2,59E-05
Slc39a6	Zinc transporter ZIP6	9,695	2,63E-05
Bpnt1	3'(2'),5'-bisphosphate nucleotidase 1	0,156	2,65E-05
Dag1	Dystroglycan 1	2,72	2,82E-05
Clu	Clusterin	5,25	2,90E-05
Hnrnpa2b1	Heterogeneous nuclear ribonucleoproteins A2/B1	0,256	2,92E-05
Tpi1	Triosephosphate isomerase	0,271	2,98E-05
Psmd10	26S proteasome non-ATPase regulatory subunit 10	0,47	3,07E-05
Ube2n	Ubiquitin-conjugating enzyme E2 N	0,259	3,08E-05
Plin3	Perilipin-3	0,109	3,25E-05
Efcab14	EF-hand calcium-binding domain-containing protein 14	3,475	3,28E-05
Raet1a	Retinoic acid early-inducible protein 1-alpha	3,489	3,40E-05
Actb	Actin, cytoplasmic 1	0,18	3,41E-05
Stmn1	Stathmin	3,457	3,57E-05
Ak2	Adenylate kinase 2, mitochondrial	0,038	3,68E-05
Adgrg6	Adhesion G-protein coupled receptor G6	3,537	4,01E-05
Lrrc59	Leucine-rich repeat-containing protein 59	0,09	4,30E-05
Psmb6	Proteasome subunit beta type-6	0,321	4,37E-05

Mdh2	Malate dehydrogenase, mitochondrial	0,184	9,26E-06
Actr3	Actin-related protein 3	0,026	9,27E-06
Ppm1g	Protein phosphatase 1G	0,216	1,03E-05
Slc39a10	Zinc transporter ZIP10	33,098	1,1E-05
Uap1l1	UDP-N-acetylhexosamine pyrophosphorylase-like protein 1	0,093	1,11E-05
Psmb1	Proteasome subunit beta type-1	0,159	1,15E-05
Tmod3	Tropomodulin-3	0,142	1,21E-05
Cdh6	Cadherin-6	2,724	1,22E-05
Actn1	Alpha-actinin-1	0,071	1,24E-05
Nptn	Neuroplastin	5,277	1,37E-05
Ostf1	Osteoclast-stimulating factor 1	0,202	1,44E-05
S100a10	Protein S100-A10	5,37	1,53E-05
Hspa4	Heat shock 70 kDa protein 4	0,238	1,53E-05

Bsg	Basigin	3,514	4,52E-05
Ruvbl1	RuvB-like 1	0,033	4,62E-05
Eef2	Elongation factor 2	0,094	4,86E-05
Eef1b	Elongation factor 1-beta	0,485	4,87E-05
Gm2a	Ganglioside GM2 activator	2,68	4,88E-05
Ahsa1	Activator of 90 kDa heat shock protein ATPase homolog 1	0,306	4,95E-05
Ablim1	Actin-binding LIM protein 1	0,262	4,95E-05
Tgoln1	Trans-Golgi network integral membrane protein 1	2,601	5,10E-05
Ints1	Integrator complex subunit 1	6,929	5,30E-05
Ran	GTP-binding nuclear protein Ran	0,229	5,30E-05
Sod3	Extracellular superoxide dismutase [Cu-Zn]	4,256	5,35E-05
Hdgf	Hepatoma-derived growth factor	0,35	5,38E-05
Bst2	Bone marrow stromal antigen 2	2,27	5,44E-05

Supplementary Figure 1

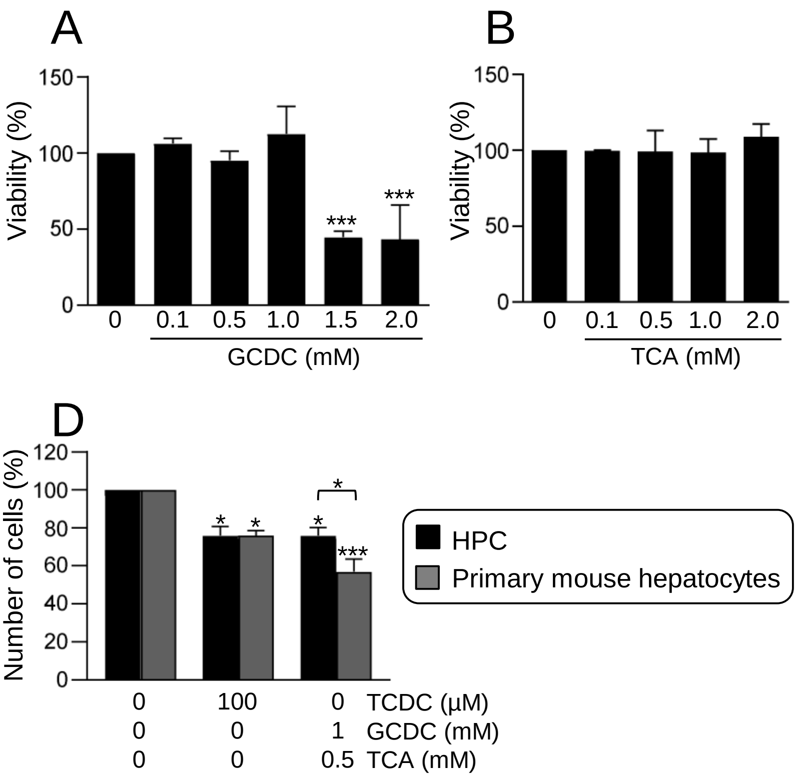

Supplementary Figure 2

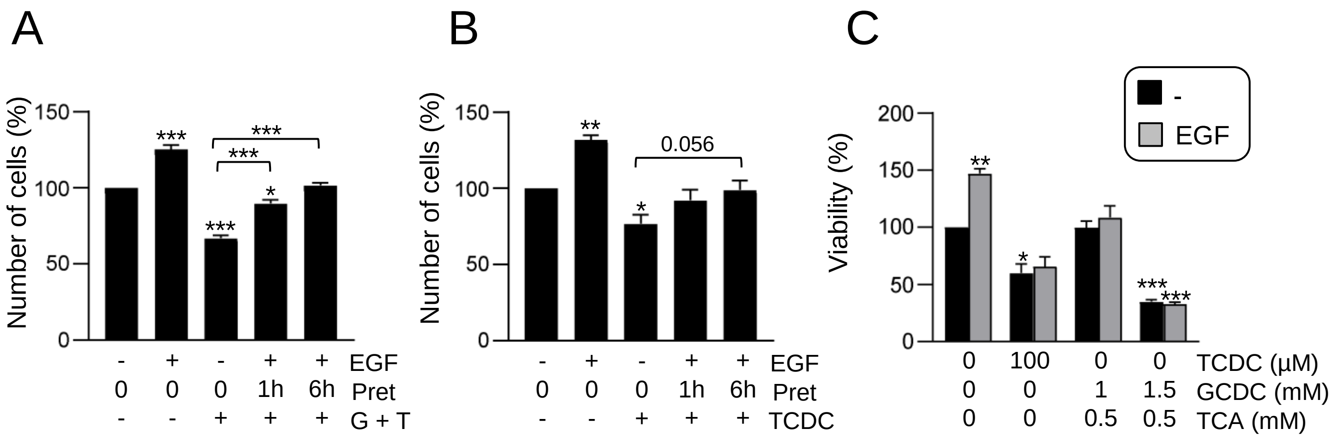

Supplementary Figure 3

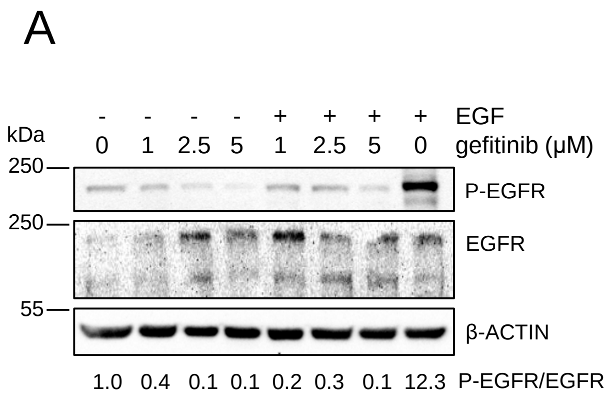

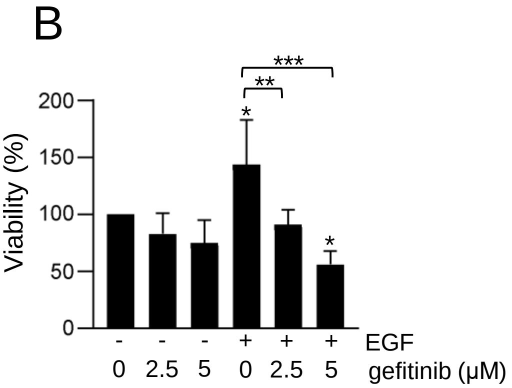

Supplementary Figure 4

A

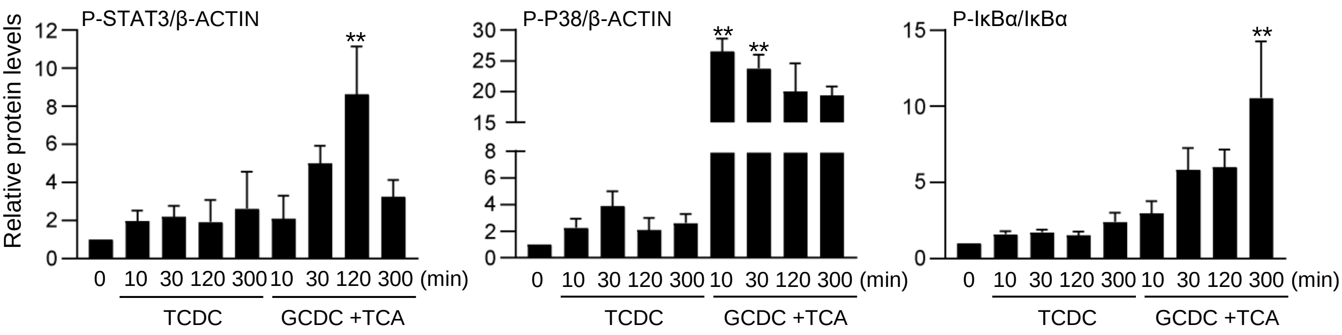

B

gene	Treatment (*fold change*)	
	**G+T**	**TCDC**
Cxcl2	3.7	4.6
Cxcl1	3.9	4.3
Il6	3.5	19.4
Tnfa	1.6	7.5
Il1b	2.1	2.4
Il4	0.9	9.7
Ccl2	1.7	7.6

C

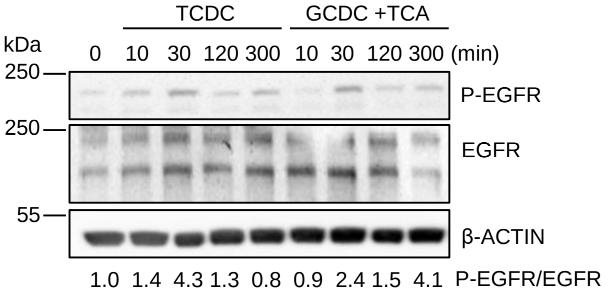

D

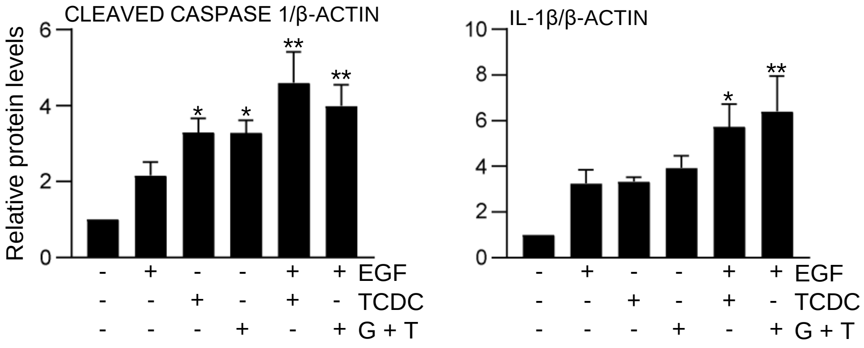

Supplementary Figure 5

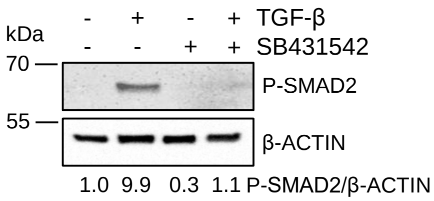

Supplementary Figure 6

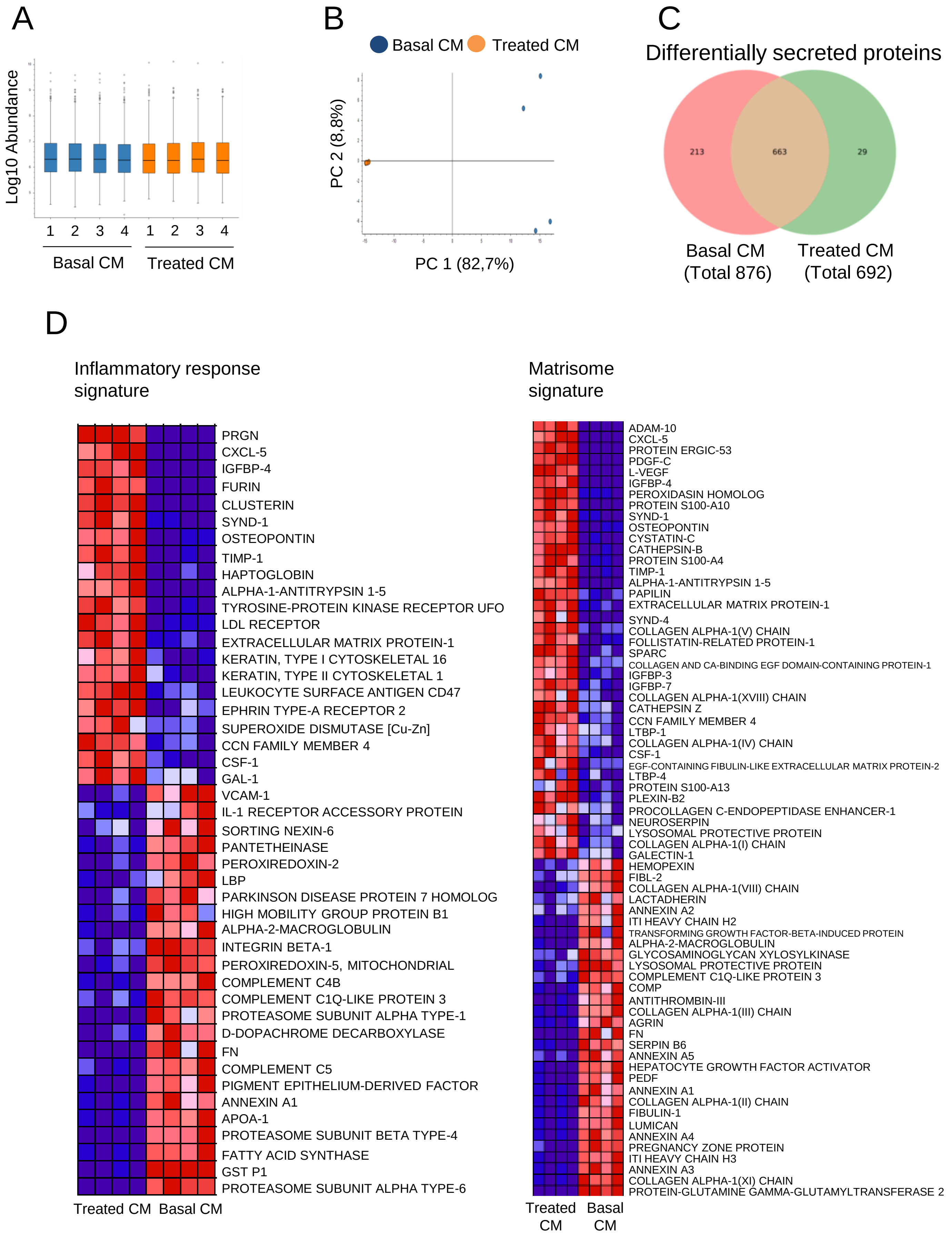

Supplement: Supplementary file 1 — Supplementary figures and tables. [file ijbsv20p2339s1.pdf]
